# Bi-Functional Composting the Sulfonic Acid Based Proton Exchange Membrane for High Temperature Fuel Cell Application

**DOI:** 10.3390/polym12051000

**Published:** 2020-04-26

**Authors:** Guoxiao Xu, Juan Zou, Zhu Guo, Jing Li, Liying Ma, Ying Li, Weiwei Cai

**Affiliations:** 1 Sustainable Energy Laboratory, Faculty of Materials Science and Chemistry, China University of Geosciences, Wuhan 430074, China; guoxiao_xu123@163.com (G.X.); Ascin123@163.com (J.Z.); zj15090728708@163.com (Z.G.); willcai1985@gmail.com (W.C.); 2 School of Chemistry and Materials Science, Guizhou Normal University, 116 Baoshan North Road, Guiyang 550001, China; 3 Research Institute for New Materials Technology, Chongqing University of Arts and Sciences, Chongqing 402160, China; leoyingchem@163.com; 4 Zhejiang Institute, China University of Geosciences, Hangzhou 311305, China

**Keywords:** bifunctionally composite, sulfonic acid based proton exchange membrane, silica nanofiber, mechanical stability, high temperature fuel cell

## Abstract

Although sulfonic acid (SA)-based proton-exchange membranes (PEMs) dominate fuel cell applications at low temperature, while sulfonation on polymers would strongly decay the mechanical stability limit the applicable at elevated temperatures due to the strong dependence of proton conduction of SA on water. For the purpose of bifunctionally improving mechanical property and high-temperature performance, Nafion membrane, which is a commercial SA-based PEM, is composited with fabricated silica nanofibers with a three-dimensional network structure via electrospinning by considering the excellent water retention capacity of silica. The proton conductivity of the silica nanofiber–Nafion composite membrane at 110 °C is therefore almost doubled compared with that of a pristine Nafion membrane, while the mechanical stability of the composite Nafion membrane is enhanced by 44%. As a result, the fuel cell performance of the silica nanofiber-Nafion composite membrane measured at high temperature and low humidity is improved by 38%.

## 1. Introduction

The excessive dependence of human on fossil fuels causes not only serious environment pollution but also global energy crises [1,2,3]. In response to these crises, new generations of sustainable energy have attracted widespread attention, such as wind energy and solar energy [4]. In order to efficiently utilize sustainable energy sources, secondary energy technologies, represented by lithium-ion battery and hydrogen energy, have to be applied as energy media [5,6,7]. As a primary segment in the hydrogen energy chain, proton-exchange membrane fuel cells (PEMFCs) have also been regard as one of the most promising green energies due to high efficiency and pollution-free properties [8,9,10]. As the core component in a PEMFC, a proton-exchange membrane (PEM) acts as a proton-transporting medium and can directly affect the performance of a PEMFC [11,12]. For numerous developed PEMs, sulfonic acid (SA) groups are the most widely used functional groups due to the great proton conductive efficiency of SA groups with the assistance of water molecules at low temperature [13,14,15]. However, enhancing the operating temperature of PEMFCs over 100 °C is a convenient strategy to improve the fuel cell performance and reduce the Pt dependence of PEMFCs [16,17,18]. Unfortunately, proton conduction performance of SA-based PEMs, represented by commercial Nafion membranes, would be strongly decayed by elevating temperature over 100 °C due to the poor water capacity of SA-based PEMs [19,20,21]. Therefore, various inorganic materials with excellent water retention capacity, including silica [22,23,24], titania [25,26], and zirconium phosphate [27], were employed to cooperate with SA groups to improve the proton conductivity of SA-based PEMs at elevated temperatures. As a result, SA-based PEMs with particulate additives usually have good high-temperature proton conductivity due to improved water retention capacity [28,29,30]. Nevertheless, poor mechanical stability of composite membranes caused by particle agglomeration or excessive swelling and bed compatibility with matrices greatly affect fuel cell performance [31]. Over the past decade, various nanofiber materials prepared by electrospinning were widely used in modification of SA-based PEMs, such as poly(vinylidene fluoride) (PVDF) [32], sulfonated polyether ether ketone (SPEEK) [33,34], and polyvinyl alcohol (PVA) [35]. Nanofibers with three-dimensional foam-like structures can not only avoid the agglomeration of particulate fillers, but also prevent excessive swelling of membranes.

Inspired by this PEM-reinforcing strategy, silica nanofibers was fabricated to bifunctionally composite a Nafion membrane, which was used as a model of SA-based PEMs, for stable application at elevated temperatures. The silica nanofibers with a porous three-dimensional network structure not only prevented excessive swelling of the membrane, but also facilitated the impregnation of a sulfonic-based conductor into the nanofibers. More importantly, the silica nanofibers without calcination treatment had abundant hydroxyl on the surface, and the nanofibers exhibited good compatibility with the SA-based proton conductor. The silica nanofibers therefore acted as a bifunctional modifier to simultaneously improve mechanical stability and high-temperature proton conductivity of the SA-based composite PEM. As a result, the proton conductivity of the silica network–Nafion composite membrane was enhanced to 0.045 S/cm at 110 °C and 60% RH with the mechanical stability improved by 44%. As a result, high-temperature fuel cell performance was strongly improved as desired.

## 2. Experimental

### 2.1. Materials

The tetrathylorthosilicate (TEOS), dimethyl sulfoxide (DMSO), hydrochioric acid and ethanol were purchased from Sinopharm Chemical Reagent Co., Ltd. (Chengdu, China). All reagents were not further treated. The Nafion 212 was supplied by Dupont Co. (Wilmington, DE, USA).

### 2.2. Preparation of Silica Nanofiber

Prior to electrospinning, a silica sol precursor solution was prepared by the following steps. Firstly, a solution of TEOS, water, ethanol, and HCl at molar ratios of 1:2:2:0.1 was prepared. Secondly, the solution was magnetically stirred for 5 h. Finally, the solution was heated on a hot plate at 80 °C, until the volume of solution was reduced to 3/8 of the original volume. The silica sol was then electrospun under the following conditions: (i) an applied voltage of 28 kV, (ii) a tip-to-collector distance of 15 cm, (iii) a flow rate of 10 μL/min.

### 2.3. Preparation of Composite Membranes

A 2 wt % Nafion membrane/dimethyl sulfoxide (DMSO) solution was prepared by the following steps. A pretreated Nafion 212 membrane was cut into small pieces. The sheared Nafion 212 membrane was dissolved in a DMSO solution, and the mixture was stirred at 150 °C under Ar atmosphere for 2 h to form a Nafion solution. A solution-casting method was used to prepare the composite membrane. Ten milliliters of the Nafion membrane/DMSO solution was added to a glass plate with a diameter of 7 cm. The plate acted as a collector, and the nanofiber was directly immersed in the Nafion solution. Then, another 10 mL of the Nafion solution was added. The mixed solution was dried in an oven at 80 °C for 24 h. Finally, the composite membrane was hot-pressed. By controlling the time of electrospinning, the contents of the silica nanofiber in the composite were 1%, 3%, and 5%, and the composite membranes were denoted as SiNF-Nafion-1%, SiNF-Nafion-3%, and SiNF-Nafion-5%, respectively. For a comparison, a pristine Nafion membrane was also prepared by the same solution-casting method.

### 2.4. Characterizations

An electrochemical workstation (Interface 1000 Gamry and CHI) was used to evaluate proton conductive performances of the membranes. With the help of a self-made test mold, all proton conduction measurements were performed in an in-plane direction by a two-probe method under high-temperature conditions and a four-probe method at full humidity. The result was calculated according to the following equation:

(1) $$\sigma =\frac{d}{RA}\;\left(S/cm\right),$$

where *σ* represents the proton conductivity of the membrane, *d* is the distance between the electrodes, *A* is the area of the membrane and *R* is the resistance (Ω) associated with the proton conductivity of the membrane obtained from the electro-chemical impedance spectroscopy (EIS) data.

Water uptake (WU) and volume swelling (VS) ratios of the membranes were measured at 25 °C. The membrane was dried under a vacuum oven at 80 °C for 24 h, and dry weight (*W*_dry_) and dry volume (*V*_dry_) were immediately recorded. Then, the membrane sample was immersed in deionized water for 24 h before the weight (*W*_wet_) and wet volume (*V*_wet_) recording. The WU and VS ratios of the membrane were calculated as shown in Equations (3) and (4):

WU= *(W*_wet_*− W*_dry_*)/W*_dry_, (2)

VS= *(V*_wet_*− V*_dry_*)/V*_dry_. (3)

The pretreated membrane was sheared into pieces, and the dry weigh was accurately weighted. The dry membrane was immersed in Fenton‘s reagent (3 ppm FeSO_4_ in 3% H_2_O_2_) at 80 °C. The oxidative stability performance was finally evaluated by the percentage of retained weights, after the membrane was treated in Fenton’s reagent for 1 and 24 h.

A scanning electron microscope (SU8010, Hitachi, Tokyo, Japan) with an energy-dispersive X-ray spectrometer (EDS) was used to study the cross-sectional morphology of the modified Nafion membrane. Small-angle X-ray scattering (SAXS) experiments were carried out on NanoSTAR with a q range from 0.007 to 0.228 Å^−1^. A NetzschSTA 409 PC TG-DTA instrument was applied for the thermal stability study for the membranes from 30 to 800 °C under nitrogen atmosphere, and the content of silica was calculated by the thermogravimetric (TG) result. Mechanical properties of all the modified Nafion membranes were evaluated by a universal tensile machine (Labthink XLW) at 25 °C. The membrane samples were cut into rectangles (size: 0.5 cm × 3 cm).

The high-temperature fuel cell was measured as follows. The electrode had a catalyst loading of 0.5 mg/cm^2^ (Pt loading: 0.2 mg/cm^2^) on both the anode and the cathode. The catalyst-coated membrane (active area: 2 cm × 2 cm) was then sandwiched between two sheets of carbon papers (TGP-H-030, Toray) after being activated in a sulfuric acid solution (0.5 M) for 12 h. PEMFC testing was carried out on a MiniTest3000 Fuel Cell Test System (TOYO Corporation) with flow rates of 0.2 and 0.5 L/min for hydrogen and oxygen, respectively.

## 3. Results and Discussion

**Figure 1:**
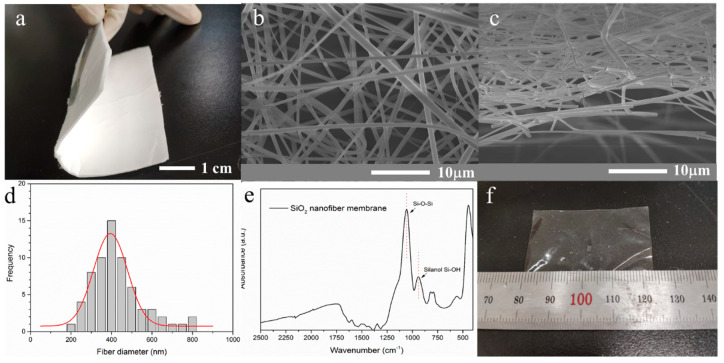
(**a**) Photograph of a pristine silica-nanofiber membrane; (**b**) surface SEM image of the pristine silica-nanofiber membrane; (**c**) cross-sectional surface SEM image of the pristine silica-nanofiber membrane; (**d**) fiber diameter distribution of the pristine silica-nanofiber membrane; (**e**) FT-IR spectrum of the pristine silica-nanofiber membrane; (**f**) photograph of the SiNF-Nafion-3% composite membrane.

Based on the electrospinning method, silica nanofibers were prepared, and the structure of THE pure thin silica-nanofiber membrane was characterized. Figure 1a is a photograph of a pristine silica-nanofiber membrane prepared by electrospinning. The nanofiber membrane was bouffant and flexible. Surface SEM images of the silica-nanofiber membrane are shown in Figure 1b,c. It can be found that the nanofibers formed a porous three-dimensional network with a porous structure. The large voids inside the three-dimensional network contributed to the impregnation of an SA-based proton-transporting conductor. According to the nanofiber diameter distribution result in Figure 1d, the silica nanofiber had an average diameter of around 390 nm. The functional groups in nanofibers were confirmed by the FT-IR spectrum of the silica-nanofiber membrane in Figure 1e, and the peaks at 1056 and 945 cm^−1^ were attributed to Si–O–Si and Si–OH vibrations, respectively. Therefore, the abundant hydroxyl on the surface of the silica nanofibers can achieve excellent interface compatibility between the nanofiber and the SA-based matrix. The silica nanofibers with a porous three-dimensional structure and abundant hydroxyl groups were successfully prepared. Then, with a Nafion membrane as a representative of the SA-based transport conductor, the silica nanofiber (SiNF)-Nafion membrane was prepared by a solution-casting method. It can be found from the photograph of a SiNF-Nafion composite membrane (SiNF-Nafion-3%) in Figure 1f that the membrane turned transparent after the composition.

**Figure 2:**
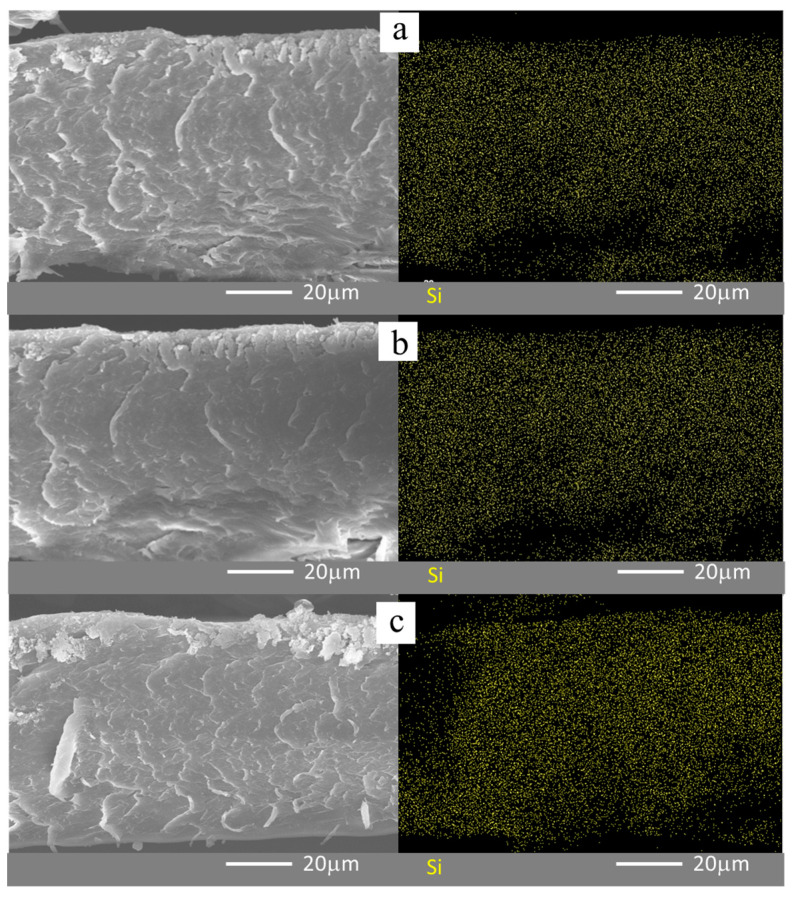
Cross-sectional SEM images and corresponding elemental maps of Si by an energy-dispersive X-ray spectrometer (EDS): (**a**) SiNF-Nafion-1% membrane; (**b**) SiNF-Nafion-3% membrane; and (**c**) SiNF-Nafion-5% membrane.

Figure 2 displays the cross-sectional SEM images of the SiNF-Nafion membranes and the corresponding elemental maps (Si) by an EDS. The cross-sections of all three SiNF-Nafion membranes remained smooth, and there was no obvious phase separation after the impregnation of Nafion polymer into the silica nanofiber. This result also demonstrated excellent compatibility between the silica nanofiber and the Nafion matrix. As shown in the elemental maps of Si, the silica nanofibers were uniformly distributed in the SiNF-Nafion membrane due to the facile impregnation of Nafion polymer. 

**Figure 3:**
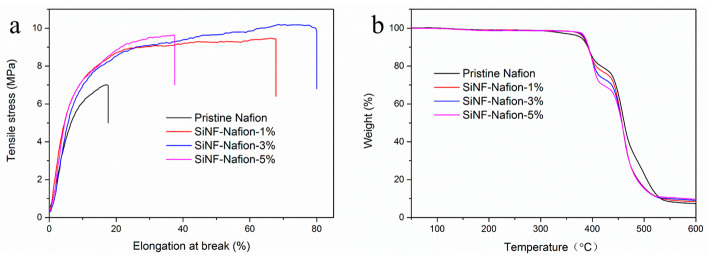
Stress-strain curves (**a**) and thermogravimetric (TG) curves (**b**) of the pristine Nafion and SiNF-Nafion membranes.

Since the silica nanofibers were confirmed to be uniformly filled into the composite membranes, the effects of nanofibers on the overall performance of the composite membranes were subsequently studied. Figure 3 compares the stress–strain and TG curves of the pristine Nafion and the SiNF-Nafion membranes. According to the stress–strain curves (Figure 3a), the tensile stresses of the three SiNF-Nafion-3% membranes were measured to be 10.2 MPa, which was 45.7% higher than that of the pristine Nafion membrane. At the same time, elongation at break of all the three SiNF-Nafion membranes was also significantly enhanced compared with that of the pristine Nafion membrane. The improved mechanical stability of the SiNF-Nafion membranes was ascribed to the uniform distribution of silica nanofibers in the composite membranes and the hydrogen bonding interactions between the hydroxyl groups on the silica nanofibers and the SA groups on the Nafion chain. With the silica nanofiber content increased to 5%, the elongation at break of the SiNF-Nafion-5% membrane was significantly reduced due to the fact that excessive silica nanofibers broke the continuity of the polymeric matrix. The mechanical stability improvement of the SiNF-Nafion membranes was also reflected by the change on the VS of the composite membranes. As shown in Table 1, the WU of the SiNF-Nafion-5% membrane was almost doubled compared with that of the pristine Nafion membrane, while the VS ratio was even lowered. The reason is that the silica nanofibers can not only efficiently retain water molecules, but also effectively limit the physical swelling of a polymeric–inorganic composite membrane. The TG curves of the pristine Nafion and SiNF-Nafion membranes shown in Figure 3b indicate that the thermal stabilities of the SiNF-Nafion membranes were not faded after the addition of silica nanofibers. At around 330 °C, the pristine Nafion membrane showed a significant mass loss, which was attributed to the decomposition of SA groups. However, for the SiNF-Nafion membranes, the decomposition temperature of the SA groups was increased to about 380 °C. This result indicated that the nanofibers had a protective effect on the SA groups. At the same time, higher residual weights of the composite membranes confirmed the successful composition of silica nanofibers with the Nafion membrane. As an important factor in evaluating the practicability of a PEM in fuel cell devices, the oxidative stability of the SiNF-Nafion membranes were found to be as good as that of the pristine Nafion membrane due to the property of silica (Table 1).

**Figure 4:**
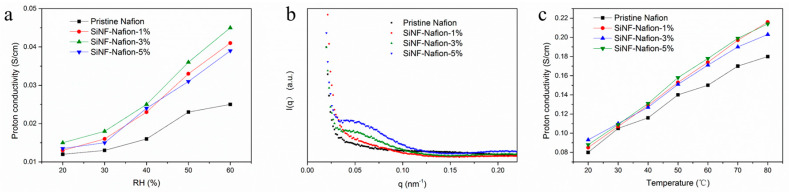
(**a**) High-temperature proton conductivity at low humidity of the pristine Nafion and SiNF-Nafion membranes; (**b**) small-angle X-ray scattering SAXS spectra of the pristine Nafion and SiNF-Nafion membranes; and (**c**) low-temperature proton conductivity at 100% relative humidity of the pristine Nafion and SiNF-Nafion membranes.

In addition to systematical stability, proton conductivity at high temperature and low humidity conditions is a great concern for applications of PEMs in high-temperature fuel cells. It can be found from Figure 4a that the SiNF-Nafion-3% membrane exhibited the highest proton conductivity at 20%–60% RH among the four membranes. At 110 °C and 20% RH, the proton conductivity of the SiNF-Nafion-3% membrane was measured to be 0.015 S/cm, which was 25% higher than that of the pristine Nafion membrane. With the humidity increased to 60% RH, the proton conductivity of the SiNF-Nafion-3% membrane was rapidly increased to 0.046 S/cm, which was 1.84 times that of the pristine Nafion membrane. This obvious increase in proton conductivity was ascribed to the great water retention capacity of the silica network uniformly composited in the membrane. However, with the nanofiber content increased to 5%, the proton conductivity of the SiNF-Nafion-5% membrane was reduced, although the increment of proton conductivity by raising the humidity was higher compared with that of the SiNF-Nafion-3% membrane. The reason is that Nafion chains impregnated into voids inside silica nanofibers still acted as a primary proton exchange medium in SiNF–Nafion composite membranes and excess silica broke the continuity of original proton conduction channels of Nafion membranes. As a result, the proton conduction efficiency was decreased, even if the water retention capacity of the membrane was enhanced for the SiNF-Nafion-5% membrane with the highest silica content. The optimum silica nanofiber content was therefore considered to be 3% for the SiNF-Nafion composite membranes. Besides, the structure changes in SA clusters may also affect proton conductivity. The SAXS spectra of the pristine Nafion and SiNF-Nafion membranes are shown in Figure 4b. Generally speaking, the SAXS peak emerging at around 0.5 nm^−1^ represents the presence of ordered SA clusters, which was the main place for proton transport in membranes. From the SAXS result, it can be concluded that no ordered SA clusters appeared in the recast Nafion membrane. With the addition of silica nanofibers, there was an obvious SAXS peak around 0.5 nm^–1^ in the SiNF-Nafion membranes. This results indicated that the ordered SA clusters were formed in the SiNF-Nafion membranes. The SA groups tended to form a cluster structure along the surface of the nanofiber due to the interaction of SA groups with hydroxyl groups on the nanofiber surface. Therefore, the synergistic effect of the excellent water retention capacity of silica nanofibers and the rearrangement of SA clusters led to an increase in the proton conductivity of the SiNF-Nafion membranes. The results were also confirmed by the 100% humidity proton conductivity of the pristine Nafion and SiNF-Nafion membranes, shown in Figure 4c. In the entire temperature range (20–60 °C), the proton conductivities of the SiNF-Nafion membranes were higher than that of the pristine Nafion membrane due to the higher proton transport efficiency of the SiNF-Nafion membranes.

**Figure 5:**
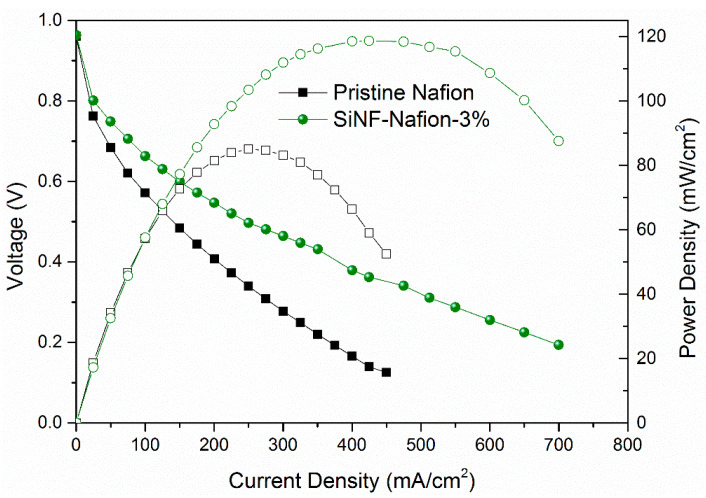
Polarization and power density curves of the pristine Nafion and SiNF-Nafion-3% membranes under 20% RH and humidified under H_2_ and O_2_ atmospheres at 110 °C.

Considering the excellent overall performance of the SiNF-Nafion-3% membrane, a fuel cell with the SiNF-Nafion-3% membrane was carried out at 110 °C and 20% RH. The polarization and power density curves of the pristine Nafion and SiNF-Nafion-3% membranes were compared in Figure 5. As expected, the SiNF-Nafion-3% membrane had a higher power density than the pristine Nafion membrane. Moreover, at the middle current range, the slope of the polarization curve of the SiNF-Nafion-3% membrane was decreased compared with that of the pristine Nafion membrane, which indicates that the internal resistance of the fuel cell was reduced. As a result, the maximum power density of the SiNF-Nafion-3% membrane was 118 mW/cm^2^, which was 38 % higher than that of the pristine Nafion membrane.

## 4. Conclusions

In summary, an SA-based proton conductor can be bifunctionally composited by a silica network. A silica nanofiber membrane with a three-dimensional porous structure can effectively retain water molecules and limit excessive water swelling of a composite membrane at the same time, because an abundant hydrogen bonding between the nanofiber and the SA-based matrix improves the mechanical strength of the composite membrane. Meanwhile, the synergistic effect of excellent water retention capacity of silica nanofiber and rearrangement of SA clusters contributes to the improvement of high-temperature proton conductivity. At 110 °C and 60% RH, the proton conductivity of a reinforced SiNF-Nafion-3% membrane is 1.84 times as high as that of the pristine Nafion membrane. As a result, the SiNF-Nafion-3% membrane exhibits great high-temperature fuel cell performance with a 118 mW/cm^2^ power density at low humidity, which is 38 % higher than that of the pristine Nafion membrane.

## Figures and Tables

**Table 1:** Water uptake, volume swelling, and oxidative stability of the pristine Nafion and SiNF-Nafion membranes.

Membrane	Water Uptake	Volume Swelling	Oxidative Stability	
			RW-1 ^1^	RW-24 ^2^
Pristine nafion	15.9%	29.3%	98.6%	98.4%
SiNF-Nafion-1%	20.4%	31.8%	98.9%	98.0%
SiNF-Nafion-3%	25.1%	28.6%	98.5%	98.1%
SiNF-Nafion-5%	28.3%	28.0%	98.8%	98.6%

^1^ Retained weight after 1 h Fenton’s reagent treatment at 80 °C. ^2^ Retained weight after 24 h Fenton’s reagent treatment at 80 °C.
